# Improvement in patient-reported outcomes and work productivity following 3-year ustekinumab or tumour necrosis factor inhibitor treatment in patients with psoriatic arthritis: results from the PsABio real-world study

**DOI:** 10.1186/s13075-023-03058-y

**Published:** 2023-06-23

**Authors:** Laure Gossec, Stefan Siebert, Paul Bergmans, Kurt de Vlam, Elisa Gremese, Beatríz Joven-Ibáñez, Tatiana V. Korotaeva, Frederic Lavie, Wim Noël, Michael T. Nurmohamed, Petros P. Sfikakis, Mohamed Sharaf, Elke Theander, Josef S. Smolen

**Affiliations:** 1 Sorbonne Université, INSERM, Institut Pierre Louis d’Epidémiologie et de Santé Publique, Paris, France; 2 Rheumatology Department, Pitié-Salpêtrière Hospital, APHP, 47–83 Boulevard de l’Hôpital, 75013 Paris, France; 3 University of Glasgow, Glasgow, UK; 4 Janssen-Cilag BV, Breda, The Netherlands; 5 University Hospitals Leuven, Leuven, Belgium; 6 Fondazione Policlinico A Gemelli-IRCCS, Università Cattolica del Sacro Cuore, Rome, Italy; 7 University Hospital, 12 de Octubre, Madrid, Spain; 8 VA Nasonova Research Institute of Rheumatology, Moscow, Russian Federation; 9 The Janssen Pharmaceutical Companies of Johnson & Johnson, Paris, France; 10 Janssen Pharmaceutica NV, Beerse, Belgium; 11 Reade and VU University Medical Center, Amsterdam, The Netherlands; 12 National and Kapodistrian University of Athens Medical School, Athens, Greece; 13 Johnson and Johnson MEA, Dubai, United Arab Emirates; 14 Janssen, Solna, Sweden; 15 Present address: Malmö University Hospital, Malmö, Sweden; 16 Medical University of Vienna, Vienna, Austria

**Keywords:** Patient-reported outcomes, Psoriatic arthritis, Real-world evidence, Tumour necrosis factor inhibitor, Ustekinumab

## Abstract

**Background:**

To evaluate the real-world effect of the IL-12/23 inhibitor ustekinumab or of a tumour necrosis factor inhibitor (TNFi) on patient-reported outcomes (PRO) and their association with effectiveness endpoints in psoriatic arthritis (PsA) patients over 3 years.

**Methods:**

In PsABio (NCT02627768), a prospective, observational study, patients with PsA that were prescribed first- to third-line ustekinumab or a TNFi, and remained on that drug for 3 years, were analysed for change in baseline in PROs (EuroQol-5 dimensions health state VAS [EQ-5D VAS], 12-item Psoriatic Arthritis Impact of Disease questionnaire [PsAID-12; range 0–10], Work Productivity and Activity Impairment for Psoriatic Arthritis questionnaire [WPAI; results expressed as a percentage for each domain]), and the association between PROs and WPAI with effectiveness endpoints, clinical disease activity index for psoriatic arthritis (cDAPSA), low disease activity (LDA)/remission, minimal disease activity (MDA) and very low disease activity (VLDA).

**Results:**

In 437 patients (mean age 49.1 years, 47.8% female), at 3 years, ustekinumab and TNFi treatment led to comparable improvements in EQ-5D VAS; mean change from baseline (95% confidence intervals [CI]) was 11.0 (6.5; 15.4) and 18.9 (14.0; 23.9), respectively. Both groups improved PsAID-12 after 3 years; mean change from baseline (95% CI) was −2.9 (−3.2; −2.5) and −3.5 (−3.9; −3.2), respectively. At baseline, due to their PsA, TNFi-treated patients had lower work productivity compared to ustekinumab-treated patients; mean productivity reduction (95% CI) was 58.8 [52.4; 65.2] and 43.3 [35.6; 51.1]. Over 3 years, TNFi-treated patients had a greater improvement in work productivity compared to ustekinumab-treated patients, ultimately leaving work productivity to be comparable between groups; mean improvement (95% CI) was 44.5% (38.4; 50.6) and 24.9% (15.8; 34.0), respectively. A similar trend was observed in activity impairment. Patients in both treatment groups who achieved effectiveness endpoints, cDAPSA LDA/remission, MDA, and VLDA had greater improvement in PROs and WPAI than patients who did not achieve these endpoints.

**Conclusions:**

At 3 years, improvements in PROs following ustekinumab or TNFi treatment were generally comparable; however, TNFi-treated patients achieved a greater improvement in work productivity, although this group started from a lower baseline. Achievement of effectiveness endpoints, independent of treatment group, also improved PROs.

**Trial registration:**

ClinicalTrials.gov, NCT02627768. Registered on 11 December 2015

## Introduction

Psoriatic arthritis (PsA) is a chronic, systemic inflammatory arthritis that affects approximately 30% of people with psoriasis, typically presenting equally in men and women between 30 and 60 years of age [1, 2]. People with PsA are commonly burdened by pain, stiffness, swollen joints, psoriasis, and psychosocial disorders, which negatively affect health-related quality of life (HRQoL) [3]. Patients with PsA suffer from sleep problems, depression, mood/behavioural changes, and reduced work productivity [3, 4]. Moreover, chronic widespread pain has a negative impact on treatment outcomes [5]. Patients with PsA have reduced HRQoL compared with the general population and have worse quality of life (QoL) than people with psoriasis alone [5].

The substantial pain and fatigue experienced by patients with PsA are significantly associated with reduced HRQoL, physical function and work productivity [6, 7]. Therefore, it is important to use patient-reported outcome (PRO) measures, in addition to physician-derived joint count or composite measure assessments, to assess physical, social and psychological functioning from the patient’s perspective in order to guide treatment decisions [8]. The increasing treatment options for PsA include tumour necrosis factor inhibitors (TNFi) and interleukin (IL)-12/IL-23 inhibitors. Ustekinumab is a fully human immunoglobulin G1 monoclonal antibody to the p40 subunit of IL-12 and IL-23 and was the first licensed non-TNFi biologic disease-modifying antirheumatic drug (bDMARD) therapy in psoriasis and PsA [9]. Additionally, ustekinumab has good efficacy against disease activity in joints and skin as well as a favourable safety profile [10–12].

While randomised clinical trials (RCTs) provide evidence on the short-term efficacy and safety of a drug, PsA is a long-term condition; thus, it is important to know the effects PsA treatments have on PROs in real-world settings over longer periods of time. Real-world data provide a greater understanding of treatment effectiveness in a patient population in routine clinical care and are, therefore, meaningful for patients and physicians making treatment decisions [12–15]. Long-term, prospective, real-world data comparing treatments for PsA with different modes of action are lacking [12].

The 6-month, 1-year and 3-year data from the prospective, observational PsABio cohort study of ustekinumab and TNFi treatment in patients with PsA indicated that persistence, effectiveness (as assessed by physician-derived joint count) and safety were generally comparable between the two treatments [16–18]. PROs reflect important functional outcomes for patients; likewise, work status/employment is essential for many patients, and 20–50% of PsA patients report unemployment due to PsA complications [19]. Real-world evidence on the long-term improvement of PROs and work disability and their link with effectiveness endpoints, low disease activity (LDA)/remission, minimal disease activity (MDA) and very low disease activity (VLDA) is still needed.

Here, we report the PRO and work productivity/activity impairment data and their association with effectiveness endpoints after 3 years of treatment with either ustekinumab or a TNFi in a real-world setting.

## Methods

### Study design

PsABio (NCT02627768) is, as previously reported, a multinational, prospective study, designed to analyse the persistence, effectiveness and safety of ustekinumab or a TNFi as a first-/second-/third-line biologic in patients with PsA as part of routine care [16–18]. Participants took part in the study for up to 3 years, with follow-up twice a year. Here we report the final 3-year analysis of patients who remained on initial treatment during the PsABio study.

### Participants

Adults (aged over 18 years) with confirmed diagnosis of PsA, as determined by a rheumatologist in line with ClASsification criteria for Psoriatic ARthritis (CASPAR), starting ustekinumab or any approved TNFi (including biosimilars) as first-, second- or third-line treatment for PsA. Participants were enrolled in 92 sites in Belgium, France, Greece, Italy, the Netherlands, the Russian Federation, Spain and the UK.

### Assessments

#### Patient-reported outcomes

##### EQ-5D-3L health state VAS

The EQ-5D-3L consists of two parts: the EuroQol-5 dimensions (EQ-5D) descriptive system and the EQ visual analogue scale (EQ VAS) [20]. The health state VAS is a vertical calibrated line bounded at 0 (worst imaginable health state) and at 100 (best imaginable health state) [21].

##### PsAID-12

The 12-item Psoriatic Arthritis Impact of Disease questionnaire (PsAID-12) is a disease-specific measure of impact of disease that uses a numerical scoring system to assess the impact of the following aspects on patients’ lives: pain, fatigue, skin problems, work and/or leisure activities, functional capacity, discomfort, sleep disturbance, coping, anger, fear and uncertainty, embarrassment and/or shame, social participation and depression. Some items are weighted differently in the scoring, which ranges from 0 (none/no difficulty/very well) to 10 (extreme/extreme difficulty/very poorly); pain, fatigue and skin problems have the highest weights [22].

##### WPAI

The Work Productivity and Activity Impairment for Psoriatic Arthritis (WPAI-PSA) questionnaire assesses work productivity loss and activity impairment due to PsA across four domains: absenteeism (work time missed), presenteeism (impairment while working), work productivity loss (work time lost due to absenteeism plus presenteeism) and activity impairment (of daily activities other than work). The WPAI-PSA consists of six questions and results are given as percentages for each domain. Higher numbers indicate greater impairment [23, 24]. Absenteeism, presenteeism and work productivity loss were assessed among employed patients only, whereas activity impairment was assessed among both employed and unemployed patients.

##### Disease activity score (cDAPSA and MDA/VLDA)

Clinical Disease Activity Index for Psoriatic Arthritis (cDAPSA) was calculated as described previously, with scores ≤14 and ≤4 denoting cDAPSA LDA and remission, respectively [25, 26]. MDA and VLDA were based on attaining five and seven, respectively, out of seven domain cut-offs, as described previously [27].

### Statistical analyses

The sponsor (Janssen Pharmaceuticals NV, Beerse, Belgium), with guidance from the authors, oversaw the development of the statistical plan, data validation and all statistical analyses.

#### Populations

The population of interest for the present analysis were patients who remained on their initial treatment line at least until day 1005 (the lower limit of the 3-year visit window), or until the end of their follow-up (for early termination—see below). Patients who were not able to reach the 3-year follow-up due to late enrolment or due to sponsor study termination were included as remainers if they were still on their initial treatment at the time the sponsor terminated the study. The study was closed prematurely due to the SARS-CoV-2 pandemic and the consequent impediment to routine patient visits, resulting in 63 patients not being able to reach the minimum of 1005 days on initial treatment due to late enrolment.

#### Analyses

As the analyses were exploratory, no predefined hypotheses were tested. No adjustment for multiplicity was applied. Hence, between-group differences and changes over time were described using 95% confidence intervals (CI) [28]. Endpoint analyses used the last observation carried forward (LOCF) for patients whose last available assessment was earlier than day 1005 and for those whose last visit was cancelled due to study termination.

EQ-5D (health state VAS), PsAID-12 total score along with PsAID skin and pain domains, and the four WPAI domains were all presented as change from baseline at 3 years (LOCF) and 95% CIs. Unadjusted observed scores with LOCF imputation for all above PROs at 3 years by achievement of effectiveness endpoints were also presented.

## Results

### Patient disposition

**Fig. 1:**
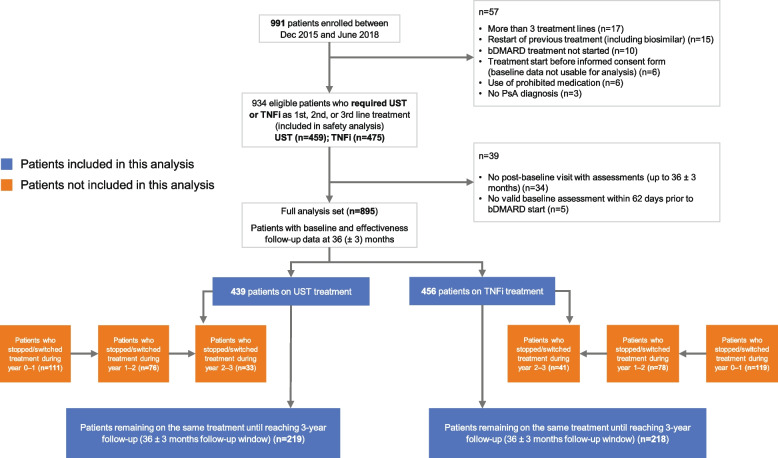
Patient population flow diagram. Abbreviations: bDMARD, biologic disease-modifying antirheumatic drug; PsA, psoriatic arthritis; TNFi, tumour necrosis factor inhibitor; UST, ustekinumab

In total, 991 participants were enrolled between December 2015 and June 2018. Of these, 895 patients had baseline and effectiveness follow-up data up to 3 years (ustekinumab *n*=439; TNFi *n*=456) and 437 patients stayed on their initial treatment for 3 years and were considered as the ‘remainer’ set included in this analysis; 219 (50.1%) ustekinumab- and 218 (49.9%) TNFi-treated (Fig. 1).

### Baseline demographics and clinical characteristics of remainers

The differences in clinical characteristics of ustekinumab and TNFi remainer groups have been described previously [17]. Patients in the ustekinumab group were older and had a greater body mass index (BMI) and more extensive skin involvement *versus* patients in the TNFi group at baseline. At baseline, a comparable number of patients in each treatment group had a Fibromyalgia Rapid Screening Tool (FiRST) score suggestive of fibromyalgia (ustekinumab; 39.0% and TNFi; 30.1%) (Table 1).

**Table 1:** Baseline demographics and clinical characteristics of patients with PsA

	**UST (*n*=219)**	**TNFi (*n*=218)**
	**Mean (SD) [95%CI] / n (%) [95%CI]**	**Mean (SD) [95%CI] / n (%) [95%CI]**
**Age, years (SD)**	**51.5 (13.0) [49.8; 53.2]**	**46.7 (12.7) [45.0; 48.4]**
Male, *n* (%)	106 (48.4) [41.6; 55.2]	122 (56.0) [49.1; 62.7]
Female, *n* (%)	113 (51.6%) [44.8; 58.4]	96 (44.0%) [37.3; 50.9]
**BMI, kg/m**^**2**^** (SD)**	**28.9 (6.4) [28.0; 29.8]**	**27.1 (5.0) [26.5; 27.8]**
Time since initial diagnosis, years (SD)	7.7 (8.5) [6.5; 8.8]	6.4 (6.7) [5.5; 7.4]
Line of bDMARD treatment, *n* (%)		
First-line	109 (49.8) [43.0; 56.6]	129 (59.2) [52.3; 65.8]
Second-line	71 (32.4) [26.3; 39.1]	69 (31.7) [25.5; 38.3]
Third-line	39 (17.8) [13.0; 23.5]	20 (9.2) [5.7; 13.8]
Cardiometabolic disease^a^, *n* (%)	95 (43.4) [36.7; 50.2]	74 (33.9) [27.7; 40.6]
PsA characteristics,* n* (%)		
Axial involvement^b^	7 (3.2) [1.3; 6.5]	7 (3.2) [1.3; 6.5]
Oligoarticular^c^	60 (27.8) [21.9; 34.3]	66 (31.0) [24.8; 37.7]
Polyarticular^d^	132 (61.1) [54.3; 67.7]	135 (63.4) [56.5; 69.9]
Dactylitis^e^, *n* (%)	46 (21.3) [16.0; 27.4]	62 (29.1) [23.1; 35.7]
Enthesitis^f^, *n* (%)	109 (50.7) [43.8; 57.6]	106 (48.8) [42.0; 55.7]
BSA, *n* (%)		
Clear/almost clear	43 (21.3) [15.9; 27.6]	58 (29.4) [23.2; 36.3]
<3% but not clear/almost clear	18 (8.9) [5.4; 13.7]	36 (18.3) [13.1; 24.4]
3–10%	72 (35.6) [29.0; 42.7]	72 (36.5) [29.8; 43.7]
**>10%**	**69 (34.2) [27.6; 41.1**]	**31 (15.7) [10.9; 21.6]**
Psoriatic nail lesions^g^, *n* (%)	117 (55.7) [48.7; 62.5]	96 (45.7) [38.8; 52.7]
cDAPSA, *n* (SD)	30.3 (21.7) [27.3; 33.3]	30.3 (20.8) [27.4; 33.2)
Swollen joint count, *n* (SD)	6.1 (8.5) [4.9; 7.2]	7.0 (8.7) [5.8; 8.3]
Tender joint count, *n* (SD)	12.7 (13.6) [10.8; 14.6]	11.8 (11.6) [10.1; 13.4]
CRP, mg/dL (SD)	1.7 (3.8) [1.1; 2.3]	1.4 (1.9) [1.1; 1.6]
FiRST score^h^		
Total score	3.5 [3.30; 3.68]	3.2 [2.99; 3.36]
Scores ≥5, %	39.0 [34.4; 43.8]	30.1 [25.8; 34.6]

Results reflect assessments for patients still under initial treatment at 3 years. Bold text indicates non-overlapping 95% CI

^a^Hypertension, myocardial infarction, congestive heart failure, stroke or transient ischemic attack, peripheral vascular disease, hyperlipidaemia, type 1 or 2 diabetes or angina pectoris

^b^Pure axial PsA is defined as having only axial involvement (presence of axial disease declared by the treating rheumatologist without a requirement for imaging)

^c^Either TJC68 and SJC66 are both non-missing and patient has <5 swollen or <5 tender joint counts or, in case TJC68 and/or SJC66 are missing, monoarticular or oligoarticular PsA is indicated by the investigator

^d^Either TJC68 and SJC66 are both non-missing and patient has ≥5 swollen and ≥5 tender joint counts or, in case TJC68 and/or SJC66 are missing, polyarticular PsA is indicated by the investigator

^e^Dactylitis presence detected by assessment of hands and feet

^f^Enthesitis presence defined as Leeds Enthesitis Index ≥0

^g^Nail lesions were evaluated by recording the total number of nails of the hands and feet with PsA involvement

^h^Based on the FAS (UST n=458 and TNFi n=471) as data were recorded at baseline only

*bDMARD*, biologic disease-modifying antirheumatic drug; *BMI*, body mass index; *BSA*, body surface area; *cDAPSA*, Clinical Disease Activity in Psoriatic Arthritis; *CI* Confidence interval, *CRP* C-reactive protein, *FAS* Full analysis set, *FiRST* Fibromyalgia Rapid Screening Tool, *PsA* Psoriatic arthritis, *SD* Standard deviation, *SJC66* 66 swollen joint count, *TJC68* 68 tender joint count, *TNFi* Tumour necrosis factor inhibitor *UST*, Ustekinumab

### Change in patient-reported outcomes following 3 years of ustekinumab or TNFi treatment (Table 2)

EQ-5D health state VAS score improved from baseline to 3 years with both treatments; mean (95% CI) change from baseline was 11.0 (6.5; 15.4) for ustekinumab and 18.9 (14.0; 23.9) for TNFi. It is worth noting that the TNFi group had a lower starting baseline value.

From baseline to 3 years, both treatments improved disease impact as measured by overall PsAID-12 score; mean change from baseline was −2.9 (95% CI −3.2; **−**2.5) and −3.5 (95% CI −3.9; −3.2), for the ustekinumab- and TNFi-treated groups, respectively. Similarly, both treatments improved the pain and skin domains of PsAID-12. Improvement in the PsAID-12 pain domain was greater in the TNFi than the ustekinumab group, even though both groups had comparable baseline values; mean change from baseline was −2.9 (95% CI −3.3; −2.5) for ustekinumab and −3.8 (95% CI −4.2; −3.4) for TNFi. Improvement in the skin domain was greater in the ustekinumab groups *versus* the TNFi group; however, a higher proportion of ustekinumab-treated patients had severe skin involvement (body surface area [BSA] >10%) and, thus, greater scope to improve. The mean change from baseline for the PsAID-12 skin domain was −3.9 (95% CI −4.4; −3.4) and −3.1 (95% CI −3.6; −2.7) for the ustekinumab- and TNFi-treated groups, respectively.

A similar proportion of patients were employed at baseline and at 3 years for both cohorts (54.5% and 55.9% for ustekinumab; 61.5% and 64.1% for TNFi, respectively). Patients in the TNFi group had higher baseline work impairment in terms of work productivity loss, presenteeism and absenteeism domains compared with patients in the ustekinumab group. This allowed patients in the TNFi group greater scope to improve in these domains, compared with the ustekinumab group. Treatment with either ustekinumab or TNFi caused a decrease in absenteeism. The TNFi treatment group had a greater numerical decrease compared to the ustekinumab treatment group. However, the 95% CIs were overlapping; mean change from baseline was −11.8% (95% CI −18.4; −5.1) and −20.8% (95% CI −27.9; −13.6) for the ustekinumab- and TNFi-treated patients, respectively. TNFi-treated patients also achieved a greater decrease in presenteeism compared to ustekinumab-treated patients; mean change from baseline was −21.6% (95% CI −28.3; −14.8) and −37.3% (95% CI −42.8; −31.8) for ustekinumab- and TNFi-treated patients, respectively. Work productivity loss decreased over the 3-year follow-up period in both treatment groups, with TNFi-treated patients demonstrating a greater decrease (as shown by non-overlapping CIs); mean change from baseline in work productivity loss was −24.9% (95% CI −34.0; −15.8) and −44.5% (95% CI −50.6; −38.4) for ustekinumab- and TNFi-treated patients, respectively. Similar to what was observed in work productivity, activity impairment with patients in the TNFi group achieved a greater decrease; mean change from baseline was −28.0% (95% CI −32.4; −23.5) and −40.7% (95% CI −44.5; −36.8) for ustekinumab- and TNFi-treated patients, respectively.

**Table 2:** Observed patient-reported outcomes at baseline and at 36 months

**Patient-reported outcomes**	**UST (*n*=219)** **Mean [95%CI]**	**TNFi (*n*=218)** **Mean [95%CI]**
EQ-5D health state: VAS score^a^		
Baseline	55.2 [52.3; 58.0]	51.1 [48.2; 54.0]
36 months	66.2 [62.4; 69.9]	70.0 [66.0; 74.1]
Change from baseline	11.0 [6.5; 15.4]	18.9 [14.0; 23.9]
PsAID-12: overall PsAID^b^		
Baseline	5.4 [5.1; 5.7]	5.3 [5.0; 5.6]
**36 months**	**2.5 [2.2; 2.8]**	**1.8 [1.5; 2.0]**
Change from baseline	−2.9 [−3.2; −2.5]	−3.5 [−3.9; −3.2]
PsAID-12: pain^c^		
Baseline	6.0 [5.6; 6.3]	6.1 [5.7; 6.4]
**36 months**	**3.0 [2.7; 3.4]**	**2.3 [2.0; 2.6]**
**Change from baseline**	−**2.9 [**−**3.3;** −**2.5]**	−**3.8 [**−**4.2;** −**3.4]**
PsAID-12: skin^d^		
Baseline	6.0 [5.6; 6.4]	4.8 [4.4; 5.2]
36 months	2.1 [1.8; 2.4]	1.6 [1.3; 1.9]
Change from baseline	−3.9 [−4.4; −3.4]	−3.1 [−3.6; −2.7]
WPAI: work time missed (absenteeism)^e^, %		
Baseline	13.2 [6.8; 19.7]	24.0 [16.4; 31.6]
36 months	1.5 [−0.1; 3.0]	3.2 [0.6; 5.8]
Change from baseline	−11.8 [−18.4; −5.1]	−20.8 [−27.9; −13.6]
WPAI: impairment while working (presenteeism)^f^, %		
**Baseline**	**37.8 [31.9; 43.7]**	**51.0 [45.4; 56.6]**
36 months	16.3 [12.2; 20.3]	13.7 [10.1; 17.2]
**Change from baseline**	−**21.6 [**−**28.3;** −**14.8]**	−**37.3 [**−**42.8;** −**31.8]**
WPAI: work productivity loss^g^, %		
**Baseline**	**43.3 [35.6; 51.1]**	**58.8 [52.4; 65.2]**
36 months	18.4 [13.5; 23.3]	14.3 [10.3; 18.3]
**Change from baseline**	−**24.9 [**−**34.0;** −**15.8]**	−**44.5 [**−**50.6;** −**38.4]**
WPAI: activity impairment^h^, %		
Baseline	53.1 [49.2; 57.0]	57.9 [54.2; 61.6]
**36 months**	**25.1 [21.6; 28.6]**	**17.3 [14.4; 20.1]**
**Change from baseline**	−**28.0 [**−**32.4;** −**23.5]**	−**40.7 [**−**44.5;** −**36.8]**

Results reflect the latest assessments for patients considered as remaining on their initial treatment through 3 years. Bold text indicates non-overlapping 95% CI. Baseline, 36-month and change from baseline values are unadjusted data with LOCF imputation. Only skin and pain domains of PsAID-12 are presented here

^a^26 patients from the UST group are missing, and 27 patients from the TNFi group are missing

^b^7 patients from the UST group are missing, and 23 patients from the TNFi group are missing

^c^7 patients from the UST group are missing, and 22 patients from the TNFi group are missing

^d^5 patients from the UST group are missing, and 21 patients from the TNFi group are missing

^e^143 patients from the UST group are missing, and 128 patients from the TNFi group are missing

^f^123 patients from the UST group are missing, and 117 patients from the TNFi group are missing

^g^143 patients from the UST group are missing, and 133 patients from the TNFi group are missing

^h^15 patients from the UST group are missing, and 21 patients from the TNFi group are missing

*CI*, confidence interval; *EQ-5D health state VAS*, EQ-5D-3L health visual analogue scale, *LOCF* Last observation carried forward, *PsAID-12* The 12-item Psoriatic Arthritis Impact of Disease questionnaire, *TNFi* Tumour necrosis factor inhibitor, *UST* Ustekinumab, *VAS* Visual analogue scale, *WPAI* Work Productivity and Activity Impairment

### Effectiveness at 3 years

Propensity score-adjusted treatment comparisons did not show differences in MDA, VLDA, cDAPSA LDA or remission between the treatment cohorts, as described previously [17].

### Relationship between PROs and effectiveness endpoints

**Fig. 2:**
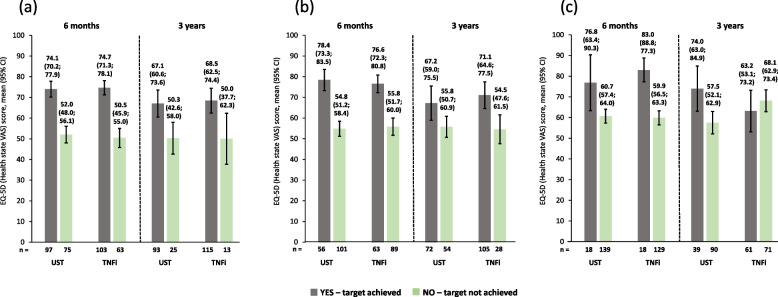
Observed EQ-5D VAS scores of patients receiving UST or TNFi by achievement of effectiveness endpoints. **a** cDAPSA LDA/remission;** b** MDA; **c** VLDA; achievement of effectiveness endpoints represented by yes**—**target achieved, or no—target not achieved. Error bars represent 95% CIs. Abbreviations: cDAPSA, Clinical Disease Activity Index for Psoriatic Arthritis; CI, confidence interval; EQ-5D, EuroQoL-5 dimensions; LDA, low disease activity; MDA, minimal disease activity; TNFi, tumour necrosis factor inhibitor; UST, ustekinumab; VAS, visual analogue scale; VLDA, very low disease activity

Patients who achieved cDAPSA LDA/remission had higher (i.e., better) EQ-5D VAS scores at 6 months than patients who did not achieve this endpoint, as shown by non-overlapping 95% CIs. This was also observed at 3 years. A similar trend was seen for patients who achieved/did not achieve MDA, although the ustekinumab group at 3 years had overlapping 95% CIs. Likewise, a similar trend was observed for patients that achieved/did not achieve VLDA, but the ustekinumab group at 6 months and the TNFi group at 3 years had overlapping 95% CIs. The scores were comparable for patients on ustekinumab or TNFi throughout (Fig. 2).

**Fig. 3:**
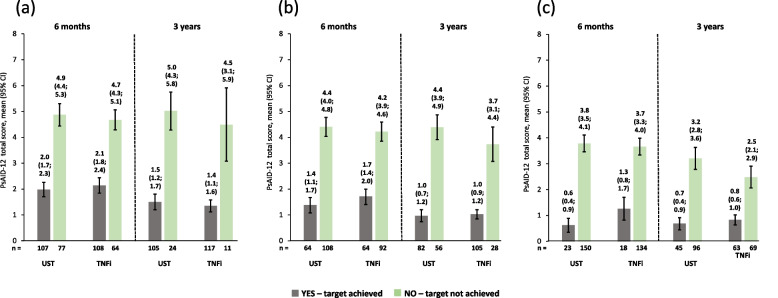
Observed PsAID-12 total scores of patients receiving UST or TNFi by achievement of effectiveness endpoints. **a** cDAPSA LDA/remission; **b** MDA; **c** VLDA; achievement of effectiveness endpoints represented by yes—target achieved, or no—target not achieved. Error bars represent 95% CIs. Abbreviations: cDAPSA, Clinical Disease Activity Index for Psoriatic Arthritis; CI, confidence interval; LDA, low disease activity; MDA, minimal disease activity; PsAID-12, The 12-item Psoriatic Arthritis Impact of Disease questionnaire; TNFi, tumour necrosis factor inhibitor; UST, ustekinumab; VLDA, very low disease activity

**Fig. 4:**
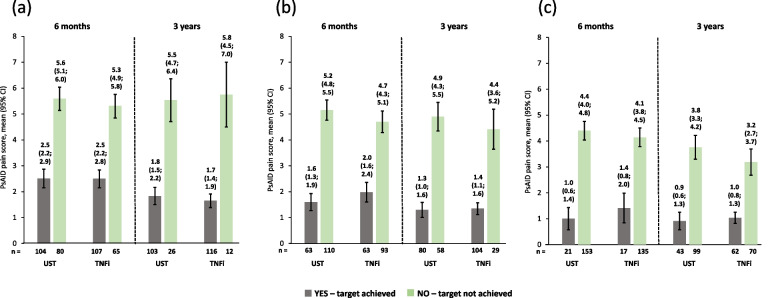
Observed PsAID pain scores of patients receiving UST or TNFi by achievement of effectiveness endpoints. **a** cDAPSA LDA/remission; **b** MDA; **c** VLDA; achievement of effectiveness endpoints represented by yes—target achieved, or no—target not achieved. Error bars represent 95% CIs. Abbreviations: cDAPSA, Clinical Disease Activity Index for Psoriatic Arthritis; CI, confidence interval; LDA, low disease activity; MDA, minimal disease activity; PsAID, Psoriatic Arthritis Impact of Disease questionnaire; TNFi, tumour necrosis factor inhibitor; UST, ustekinumab; VLDA, very low disease activity

**Fig. 5:**
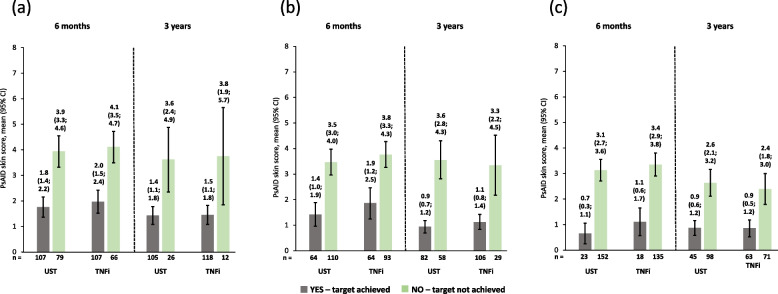
Observed PsAID skin scores of patients receiving UST or TNFi by achievement of effectiveness endpoints. **a** cDAPSA LDA/remission; **b** MDA; **c** VLDA; achievement of effectiveness endpoints represented by yes—target achieved or no—target not achieved. Error bars represent 95% CIs. Abbreviations: cDAPSA, Clinical Disease Activity Index for Psoriatic Arthritis; CI, confidence interval; LDA, low disease activity; MDA, minimal disease activity; PsAID, Psoriatic Arthritis Impact of Disease questionnaire; TNFi, tumour necrosis factor inhibitor; UST, ustekinumab; VLDA, very low disease activity

The observed PsAID-12 scores were much lower (i.e., better) for patients who achieved cDAPSA LDA/remission at 6 months compared with those who did not achieve this endpoint, as shown by non-overlapping 95% CIs. These improvements were maintained until 3 years. Similar trends at both timepoints were seen for patients who achieved/did not achieve MDA and those who achieved/did not achieve LDA. Patients on ustekinumab and those on TNFi had comparable PsAID-12 scores at 6 months and 3 years (Fig. 3). Similar trends were observed for PsAID pain and skin domains in both ustekinumab- and TNFi-treated patients (Figs. 4 and 5).

**Fig. 6:**
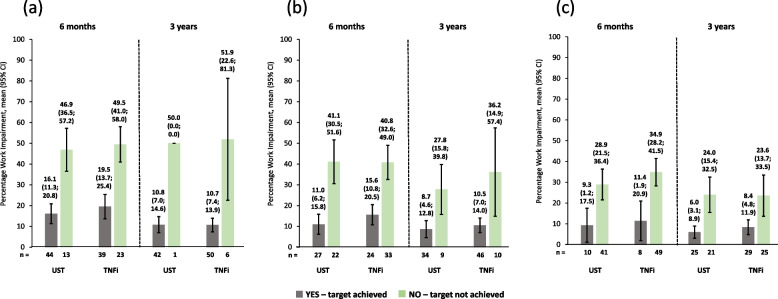
Observed percentage work impairment of patients receiving UST or TNFi by achievement of effectiveness endpoints. **a** cDAPSA LDA/remission; **b** MDA; **c** VLDA; achievement of effectiveness endpoints represented by yes—target achieved, or no—target not achieved. Error bars represent 95% CIs. Work impairment due to PsA is calculated for employed patients only, calculated by work time lost due to absenteeism plus presenteeism, expressed as a percentage. Abbreviations: cDAPSA, Clinical Disease Activity Index for Psoriatic Arthritis; CI, confidence interval; LDA, low disease activity; MDA, minimal disease activity; PsA, psoriatic arthritis; TNFi, tumour necrosis factor inhibitor; UST, ustekinumab; VLDA, very low disease activity

**Fig. 7:**
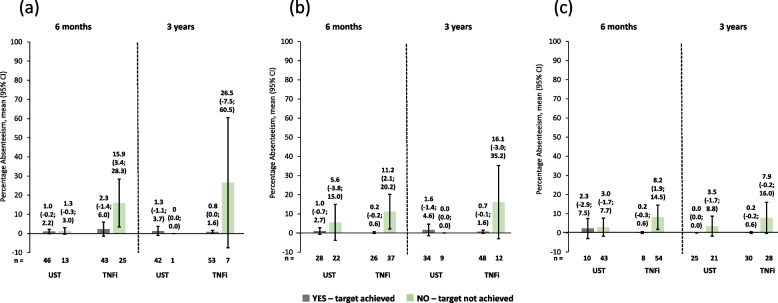
Observed percentage absenteeism of patients receiving UST or TNFi by achievement of effectiveness endpoints. **a** cDAPSA LDA/remission; **b** MDA; **c** VLDA; achievement of effectiveness endpoints represented by yes—target achieved, or no—target not achieved. Error bars represent 95% CIs. Absenteeism due to PsA is calculated for employed patients only, measured as work time missed (hours) in the last 7 days, expressed as a percentage. Abbreviations: cDAPSA, Clinical Disease Activity Index for Psoriatic Arthritis; CI, confidence interval; LDA, low disease activity; MDA, minimal disease activity; PsA, psoriatic arthritis; TNFi, tumour necrosis factor inhibitor; UST, ustekinumab; VLDA, very low disease activity

**Fig. 8:**
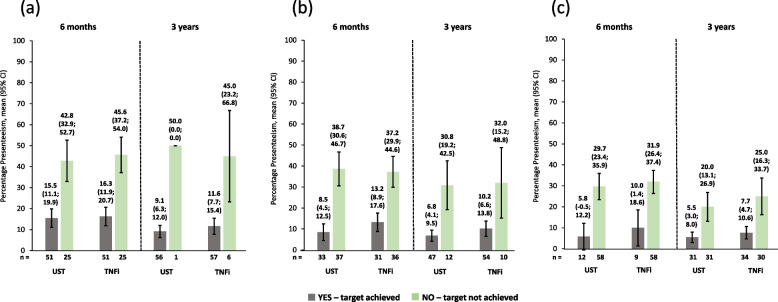
Observed percentage presenteeism of patients receiving UST or TNFi by achievement of effectiveness endpoints. **a** cDAPSA LDA/remission; **b** MDA; **c** VLDA; achievement of effectiveness endpoints represented by yes—target achieved, or no—target not achieved. Error bars represent 95% CIs. Presenteeism due to PsA is calculated for employed patients only, measured on a scale from 0 = ‘least’ to 10 = ‘worst’, expressed as a percentage. Abbreviations: cDAPSA, Clinical Disease Activity Index for Psoriatic Arthritis; CI, confidence interval; LDA, low disease activity; MDA, minimal disease activity; PsA, psoriatic arthritis; TNFi, tumour necrosis factor inhibitor; UST, ustekinumab; VLDA, very low disease activity

**Fig. 9:**
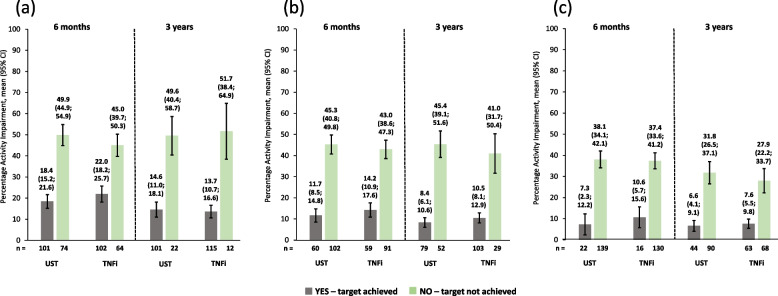
Observed percentage activity impairment of patients receiving UST or TNFi by achievement of effectiveness endpoints. **a** cDAPSA LDA/remission; **b** MDA; **c** VLDA; achievement of effectiveness endpoints represented by yes—target achieved, or no—target not achieved. Error bars represent 95% CIs. The percentage activity impairment due to PsA is calculated for both employed and unemployed patients. Abbreviations: cDAPSA, Clinical Disease Activity Index for Psoriatic Arthritis; CI, confidence interval; LDA, low disease activity; MDA, minimal disease activity; PsA, psoriatic arthritis; TNFi, tumour necrosis factor inhibitor; UST, ustekinumab; VLDA, very low disease activity

Work impairment scores were lower (i.e., better) for patients achieving cDAPSA LDA/remission or MDA or VLDA at 6 months *versus* those who did not achieve these endpoints, as shown by non-overlapping 95% CIs. Similar improvements were seen at 3 years. Again, in this group, ustekinumab and TNFi produced comparable work impairment scores throughout (Fig. 6). Absenteeism and presenteeism scores (Figs. 7 and 8) were more variable, probably due to low numbers of patients, but similar trends were observed. It is worth noting that in the absenteeism scores most 95% CIs were overlapping. Similar changes to those seen in work productivity loss were observed in activity impairment scores (Fig. 9).

## Discussion

In the PsABio real-world study, PROs improved from baseline to 3 years in a broadly similar manner for both ustekinumab and TNFi treatment cohorts. Ustekinumab has previously been shown to improve PROs in patients with PsA in a RCT setting. A post hoc analysis of PSUMMIT-1 (*n*=615) and PSUMMIT-2 (*n*=312) RCTs showed that at 24 weeks, ustekinumab treatment resulted in significant improvements in PROs *versus* placebo in three antecedent-exposure populations (methotrexate [MTX] anti-TNF naïve; MTX experienced, biologic agent naïve; anti-TNF experienced with/without MTX). These findings indicated that significant improvements with ustekinumab were observed, regardless of previous treatments for PsA [29].

Work productivity improved with both ustekinumab and TNFi treatments in the PsABio study, with more improvement from baseline observed in the TNFi-treated group, who started from a lower baseline. The scores rapidly decreased from baseline to 6 months and then continued to steadily decrease through the 3 years of treatment. Non-overlapping CIs in presenteeism, work productivity loss and activity impairment domains suggest that TNFi treatment may lead to a greater improvement in work productivity and activity impairment, although the non-randomised setting, differential baseline work impairment and patient heterogeneity impact interpretation and preclude firm conclusions.

Real-world data on the impact of different kinds of treatment on PROs in PsA is limited. However, a recent RWE study that included 120 PsA patients receiving TNFi treatment showed improvements in work productivity scores and PROs over 9 months of treatment [30]. These patients demonstrated a significant decrease in all WPAI domain scores as well as significant improvements to other QoL markers such as the health assessment questionnaire-disability index after TNFi treatment. The improvements we have seen with both ustekinumab and TNFi over a 3-year period are consistent with this previous study focusing on TNFis over 9 months.

In addition, our results are in line with what has been shown for bDMARDs, which have an alternative mechanism of action. In the 2-year follow-up of the Phase 3 SPIRIT-P2 study, patients receiving ixekizumab, an IL-17A inhibitor, showed sustained improvements in percentage of presenteeism, overall work impairment and activity impairment, along with improvements in other QoL measures [31]. Of note, patients in the SPIRIT-P2 trial were either intolerant of or had inadequate response to TNFi [31]. The SPIRIT-P1 trial which recruited patients with PsA who were bDMARD naïve also showed significant improvements in PROs and work productivity with ixekizumab treatment [32]. In a post hoc analysis of the Phase 3 FUTURE 5 trial, treatment with secukinumab, an IL-17A inhibitor, resulted in early, statistically significant and clinically meaningful sustained improvements in PROs *versus* placebo in patients with PsA, regardless of prior TNFi use [33].

In PsABio, treatment with ustekinumab and TNFi resulted in similar proportions of patients with PsA achieving the effectiveness endpoints of cDAPSA LDA/remission, MDA and VLDA at 1 and 3 years [17, 18]. Patients who achieved the effectiveness endpoints, including VLDA, at 3 years generally had greatly improved EQ-5D VAS, PsAID-12 and work productivity loss/activity impairment scores compared with patients who did not achieve them. Absenteeism/presenteeism scores followed a similar trend to the other WPAI domains; however, these results were more variable. It is difficult to draw strong conclusions due to the low numbers of patients who did not achieve effectiveness endpoints with either treatment at the end of 3 years. These findings are consistent with the results from the PsABio 6-month interim analysis that showed patients achieving LDA had associated improvement in QoL and disease impact [16]. Interestingly, there were patients who did not achieve effectiveness endpoints at 6 months or 3 years but remained on one treatment for the duration of the study. Due to the observational nature of this study, it is possible that some patients were not achieving effectiveness endpoints, but, nevertheless, the treating physician observed some benefit for the patient to continue ustekinumab or TNFi treatment or some other factors made switching bDMARD less desirable. Some patients, particularly those receiving ustekinumab, were on a later line of treatment. Due to this, their ability to switch to a bDMARD with a different mechanism of action could have been limited, and, thus, they remained on their current treatment. Patients achieving cDAPSA LDA/remission, MDA and VLDA had greater PRO and WPAI scores compared to patients who did not achieve effectiveness endpoints. Of note, VLDA is a hard-to-achieve high hurdle endpoint, but treatment towards reaching the goal of achieving VLDA did not seem to make an additional difference to patients’ QoL beyond MDA. Achieving VLDA may also be associated with a high number of adverse events due to robust treatment and may not be appropriate for all patients, particularly those with high levels of existing structural damage and functional disability [34]. In the open-label, randomised controlled TICOPA trial of 206 patients, although tight control of PsA improved joint and skin outcomes for newly diagnosed PsA patients, these patients reported adverse events and serious adverse events more frequently [35].

The present analysis is the first to report PROs and their association with achievement of effectiveness endpoints in PsA in a real-world population receiving ustekinumab or a TNFi. The association between PRO improvement and improvements in effectiveness endpoints, taken together with the existing literature, suggests that achieving LDA, regardless of the therapy, is likely to be the key contributor to improved HRQoL and PROs.

This real-world data generated by the PsABio study informs clinicians of the long-term benefits on PROs of ustekinumab and TNFi treatment in routine clinical care. However, real-world data are also valuable to public and private payers for decision-making. Real-world studies are valuable for filling evidence gaps not addressed by RCTs, such as long-term effectiveness, safety profile and PROs in real-world settings [36].

As reported previously, PsABio consists of a large, prospectively followed population with PsA receiving bDMARDs with two different modes of action [16–18]. The limitation, however, is the non-randomised nature of the study, which means that the treatment groups need to be balanced using propensity-score adjustment due to selection bias. The influence of factors such as fibromyalgia on different PROs and WPAI should be investigated in the future.

## Conclusion

In conclusion, the results from the 3-year PsABio study demonstrated that, generally, ustekinumab and TNFi treatment led to an improvement in PROs. In certain PROs, TNFi-treated patients showed greater improvement compared with ustekinumab-treated patients but may have been confounded by differential baseline factors influencing treatment decisions. Patients achieving effectiveness endpoints had improved PROs, independent of treatment group. These findings may be useful for physicians in aiding treatment decisions in clinical practice.

## Data Availability

Access to anonymised, individual participant-level data will not be provided for this trial as it meets one or more of the exceptions described on https://yoda.yale.edu/ under ‘Data Use Agreement - Janssen Pharmaceuticals DUA’.

## Abbreviations

bDMARD: Biologic disease-modifying antirheumatic drug
BMI: Body mass index
BSA: Body surface area
cDAPSA: Clinical disease activity index for psoriatic arthritis
CI: Confidence intervals
EQ-5D: EuroQol-5 dimensions
EQ VAS: EQ visual analogue scale
HRQoL: Health-related quality of life
IL: Interleukin
LDA: Low disease activity
LOCF: Last observation carried forward
MDA: Minimal disease activity
MTX: Methotrexate
PRO: Patient-reported outcome
PsA: Psoriatic arthritis
PsAID-12: 12-item Psoriatic Arthritis Impact of Disease Questionnaire
QoL: Quality of life
RCT: Randomised clinical trials
TNFi: Tumour necrosis factor inhibitors
VLDA: Very low disease activity
WPAI: Work Productivity and Activity Impairment
WPAI-PSA: Work Productivity and Activity Impairment for Psoriatic Arthritis

## References

[1] FitzGerald O, Ogdie A, Chandran V, Coates LC, Kavanaugh A, Tillett W, Leung YY, deWit M, Scher JU, Mease PJ (2021). Psoriatic arthritis. Nat Rev Dis Primers.

[2] Rech J, Sticherling M, Stoessel D, Biermann MHC, Haberle BM, Reinhardt M (2020). Psoriatic arthritis epidemiology, comorbid disease profiles and risk factors: results from a claims database analysis. Rheumatol Adv Pract.

[3] Haugeberg G, Michelsen B, Kavanaugh A. Impact of skin, musculoskeletal and psychosocial aspects on quality of life in psoriatic arthritis patients: a cross-sectional study of outpatient clinic patients in the biologic treatment era. RMD Open. 2020;6(1):e001223. 10.1136/rmdopen-2020-001223 PMC7299507 32409518

[4] Haugeberg G, Lund Nilsen TI, Kavanaugh A, Thomsen RS, Gulati AM, Hoff M (2021). Physical and psychosocial burden of psoriatic arthritis: longitudinal data from a population-based study in Norway. Arthritis Care Res (Hoboken).

[5] Batko B (2020). Patient-centered care in psoriatic arthritis-a perspective on inflammation, disease activity, and psychosocial factors. J Clin Med.

[6] Husni ME, Merola JF, Davin S (2017). The psychosocial burden of psoriatic arthritis. Semin Arthritis Rheum.

[7] Conaghan PG, Alten R, Deodhar A, Sullivan E, Blackburn S, Tian H, Gandhi K, Jugl SM, Strand V (2020). Relationship of pain and fatigue with health-related quality of life and work in patients with psoriatic arthritis on TNFi: results of a multi-national real-world study. RMD Open.

[8] Kitchen H, Cordingley L, Young H, Griffiths CE, Bundy C (2015). Patient-reported outcome measures in psoriasis: the good, the bad and the missing!. Br J Dermatol.

[9] Davari P, Leo MS, Kamangar F, Fazel N (2014). Ustekinumab for the treatment of psoriatic arthritis: an update. Clin Cosmet Investig Dermatol.

[10] McInnes IB, Kavanaugh A, Gottlieb AB, Puig L, Rahman P, Ritchlin C, Brodmerkel C, Li S, Wang Y, Mendelsohn AM (2013). Efficacy and safety of ustekinumab in patients with active psoriatic arthritis: 1 year results of the phase 3, multicentre, double-blind, placebo-controlled PSUMMIT 1 trial. Lancet.

[11] Ritchlin C, Rahman P, Kavanaugh A, McInnes IB, Puig L, Li S, Wang Y, Shen Y-K, Doyle MK, Mendelsohn AM (2014). Efficacy and safety of the anti-IL-12/23 p40 monoclonal antibody, ustekinumab, in patients with active psoriatic arthritis despite conventional non-biological and biological anti-tumour necrosis factor therapy: 6-month and 1-year results of the phase 3, m. Ann Rheum Dis.

[12] Menter A, Papp KA, Gooderham M, Pariser DM, Augustin M, Kerdel FA, Fakharzadeh S, Goyal K, Calabro S, Langholff W (2016). Drug survival of biologic therapy in a large, disease-based registry of patients with psoriasis: results from the Psoriasis Longitudinal Assessment and Registry (PSOLAR). J Eur Acad Dermatol Venereol.

[13] Vandendorpe A-S, de Vlam K, Lories R (2019). Evolution of psoriatic arthritis study patient population characteristics in the era of biological treatments. RMD Open.

[14] Ogdie A, Coates L (2017). The changing face of clinical trials in psoriatic arthritis. Curr Rheumatol Rep.

[15] Blonde L, Khunti K, Harris SB, Meizinger C, Skolnik NS (2018). Interpretation and impact of real-world clinical data for the practicing clinician. Adv Ther.

[16] Smolen JS, Siebert S, Korotaeva TV, Selmi C, Bergmans P, Gremese E, Joven-Ibanez B, Katsifis G, Noel W, Nurmohamed MT (2021). Effectiveness of IL-12/23 inhibition (ustekinumab) versus tumour necrosis factor inhibition in psoriatic arthritis: observational PsABio study results. Ann Rheum Dis.

[17] Gossec L, Siebert S, Bergmans P (2023). Long-term effectiveness and persistence of ustekinumab and TNF inhibitors in patients with psoriatic arthritis: final 3-year results from the PsABio real-world study. Ann Rheum Dis.

[18] Gossec L, Siebert S, Bergmans P, de Vlam K, Gremese E, Joven-Ibanez B, Korotaeva TV, Lavie F, Noel W, Nurmohamed MT (2022). Persistence and effectiveness of the IL-12/23 pathway inhibitor ustekinumab or tumour necrosis factor inhibitor treatment in patients with psoriatic arthritis: 1-year results from the real-world PsABio Study. Ann Rheum Dis.

[19] Tillett W (2012). de-Vries C, McHugh NJ: Work disability in psoriatic arthritis: a systematic review. Rheumatology (Oxford).

[20] EuroQol Group (1990). EuroQol–a new facility for the measurement of health-related quality of life. Health Policy.

[21] Whynes DK (2008). TOMBOLA Group: Correspondence between EQ-5D health state classifications and EQ VAS scores. Health Qual Life Outcomes.

[22] Gossec L, de Wit M, Kiltz U, Braun J, Kalyoncu U, Scrivo R, Maccarone M, Carton L, Otsa K, Sooaar I (2014). A patient-derived and patient-reported outcome measure for assessing psoriatic arthritis: elaboration and preliminary validation of the Psoriatic Arthritis Impact of Disease (PsAID) questionnaire, a 13-country EULAR initiative. Ann Rheum Dis.

[23] Reilly MC, Zbrozek AS, Dukes EM (1993). The validity and reproducibility of a work productivity and activity impairment instrument. Pharmacoeconomics.

[24] Tang K, Beaton DE, Boonen A, Gignac MA, Bombardier C. Measures of work disability and productivity: Rheumatoid Arthritis Specific Work Productivity Survey (WPS-RA), Workplace Activity Limitations Scale (WALS), Work Instability Scale for Rheumatoid Arthritis (RA-WIS), Work Limitations Questionnaire (WLQ), and Work Productivity and Activity Impairment Questionnaire (WPAI). Arthritis Care Res (Hoboken). 2011;63 Suppl 11:S337–49. 10.1002/acr.20633. 10.1002/acr.20633 22588755

[25] Schoels MM, Aletaha D, Alasti F, Smolen JS (2016). Disease activity in psoriatic arthritis (PsA): defining remission and treatment success using the DAPSA score. Ann Rheum Dis.

[26] Aletaha D, Alasti F, Smolen JS (2017). Disease activity states of the DAPSA, a psoriatic arthritis specific instrument, are valid against functional status and structural progression. Ann Rheum Dis.

[27] Coates LC, Helliwell PS (2016). Defining low disease activity states in psoriatic arthritis using novel composite disease instruments. J Rheumatol.

[28] Harrington D, D'Agostino RB, Gatsonis C, Hogan JW, Hunter DJ, Normand ST, Drazen JM, Hamel MB (2019). New Guidelines for Statistical Reporting in the Journal. N Engl J Med.

[29] Rahman P, Puig L, Gottlieb AB, Kavanaugh A, McInnes IB, Ritchlin C, Li S, Wang Y, Song M, Mendelsohn A (2016). Ustekinumab treatment and improvement of physical function and health-related quality of life in patients with psoriatic arthritis. Arthritis Care Res (Hoboken).

[30] Karadag O, Dalkilic E, Ayan G, Kucuksahin O, Kasifoglu T, Yilmaz N, Koca SS, Yazisiz V, Erten PT, Sayarlioglu M (2022). Real-world data on change in work productivity, activity impairment, and quality of life in patients with psoriatic arthritis under anti-TNF therapy: a postmarketing, noninterventional, observational study. Clin Rheumatol.

[31] Turkiewicz A, Gellett AM, Kerr L, Birt J, Gratacos J. Long-term effect of ixekizumab on patient-reported outcomes in patients with Psa and inadequate response to TNF inhibitors: 2-year follow-up from a phase 3 study [abstract]. Arthritis Rheumatol. 2018;70(suppl 10). https://acrabstracts.org/abstract/long-term-effect-of-ixekizumab-on-patient-reported-outcomes-in-patients-with-psa-and-inadequate-response-to-tnf-inhibitors-2-year-follow-up-from-a-phase-3-study/. Accessed 9 Mar 2022.

[32] Gottlieb AB, Strand V, Kishimoto M, Mease P, Thaci D, Birt J, Lee CH, Shuler CL, Lin CY, Gladman DD (2018). Ixekizumab improves patient-reported outcomes up to 52 weeks in bDMARD-naive patients with active psoriatic arthritis (SPIRIT-P1). Rheumatology (Oxford).

[33] Strand V, Kaeley GS, Bergman MJ, Gladman D, Coates L, Sherif B, Hur P, Parikh B, Gilloteau I, Mease P (2022). The effect of secukinumab on patient-reported outcomes in patients with active psoriatic arthritis in a randomised phase 3 trial. Lancet Rheumatol.

[34] Coates LC, Smolen JS, Mease PJ, Husni ME, Merola JF, Lespessailles E, Kishimoto M, Macpherson L, Bradley AJ, Bolce R (2022). Comparative performance of composite measures from two phase III clinical trials of ixekizumab in psoriatic arthritis. RMD Open.

[35] Coates LC, Moverley AR, McParland L, Brown S, Navarro-Coy N, O'Dwyer JL, Meads DM, Emery P, Conaghan PG, Helliwell PS (2015). Effect of tight control of inflammation in early psoriatic arthritis (TICOPA): a UK multicentre, open-label, randomised controlled trial. Lancet.

[36] Roberts MH, Ferguson GT (2021). Real-World Evidence: Bridging Gaps in Evidence to Guide Payer Decisions. Pharmacoecon Open.

